# Advantages in orthopaedic implant infection diagnostics by additional analysis of explants

**DOI:** 10.1007/s00264-025-06424-4

**Published:** 2025-02-19

**Authors:** Arnold J. Suda, Thomas Miethke, Nadine Landua, Udo Obertacke

**Affiliations:** 1 Institute of Medical Microbiology and Hygiene, University Medical Centre Mannheim, Medical Faculty Mannheim, Heidelberg University, Theodor-Kutzer-Ufer 1-3, 68167 Mannheim, Germany; 2 Department of Orthopaedics and Trauma Surgery, University Medical Centre Mannheim, Medical Faculty Mannheim, Heidelberg University, Theodor-Kutzer-Ufer 1-3, 68167 Mannheim, Germany

**Keywords:** Orthopaedic implant infection diagnostics, Implant surface, Implant removal

## Abstract

**Purpose:**

Implant-associated infections are the most challenging complication in orthopaedics and trauma surgery as they often lead to long courses of illness and are a financial burden for the healthcare system. There is a need for fast, simple, and cheap identification of pathogens but the ideal detection method was not found yet. The work aims to test whether the detection of pathogens culturing the removed implant is more successful than from simultaneously taken tissue samples or punction fluid.

**Methods:**

Implants were removed due to infection, irritation, or loosening. Tissue samples and joint fluids were processed for bacterial growth in sterile conditions. Samples were incubated and checked for growth. Bacterial identification and antibiotic sensitivity testing were performed. Data were anonymized, and statistical analysis was done using Excel and SAS, employing tests like Shapiro-Wilk, Mann-Whitney-U, and Kruskal-Wallis. Ethical approval was obtained for this study.

**Results:**

Between February 2018 and April 2019, a total of 163 patients (175 cases) underwent orthopaedic implant removal for various reasons. 30 cases were not usable or analyzable due to missing or damaged reference material, so 145 cases could be evaluated due to study protocol.

The range of detected bacteria was as expected and included low-virulent bacteria such as *Micrococcus luteus* and *Corynebacteria*. Pathogen detection by culture of the the explant´s was more sensitive (84.83%) than pathogen detection from tissue samples and punction fluid (64.14%, p<0.0001). Comorbidities did not play any role in the quality of detection but prior antibiotic treatment did influence the results of tissue diagnostics.

**Conclusion:**

This study showed with a higher frequency of bacterial detection of orthopedic explant´s surface compared to tissue samples or punction fluid. This may reduce the number of samples and cost but enhances the quality of orthopaedic implant-related infection diagnostics.

## Introduction

Orthopaedic implants are removed for several reasons such as infection, pain, or mechanical issues. Infection is a main cause but there is a high number of occult and low-grade-infection which are difficult to find. Detection of bacteria after orthopedic implant removal can be influenced by prior antibiotic treatment and the methods used for it. As treatment of orthopaedic implant-related infection (OII) is not rare but expensive and harms patient´s quality of life, fast and correct diagnosis and treatment are crucial [1–4]. Infection after orthopaedic implant implantation can be caused by perioperative contamination, haematogenous infection, or *per continuitatem* [5–7]. Biofilm on the implant´s surface is often present [8, 9]. Different classification systems define acute and chronic infections [8, 10, 11]. There are many known risk factors for OII, such as comorbidities, prolonged operation time, smoking, and revision surgery [9, 10, 12–18]. Coagulase-negative staphylococci such as *Staphylococcus epidermidis*, part of the natural skin microbiome, are often causal for orthopaedic implant-related infections. If they occur in joints or on implants, they are pathogens [6]. Together with *Staphylococcus aureus*, coagulase-negative staphylococci are responsible for 50% of OII [5]. The other 50% are caused by *Streptococci, Enterococci,* Gram-negative rods, anaerobes*, Candida*, and rare pathogens [6]. The presence of low-virulent bacteria such as coagulase-negative staphylococci, anaerobe *Cutibacterium (Propionibacterium) acnes*, and Gram-positive *Corynebacteria* are often caused by contamination and may lead to a chronic condition and slow development of infection. High-virulent bacteria such as *Staphylococcus aureus, Escherichia coli,* and *Streptococci* may lead to early and fulminant infection up to sepsis [6, 19]. In 10–30%, multiple bacteria with a minimum of two pathogens are detected [5]. Biofilm of the implant´s surface is the main cause of bacterial infection [3, 20]. A matrix of extra-cellular polymer substances (EPS) surrounds the implant and safes bacteria from antibiotics and the body´s defense cells [3, 21, 22]. There are different options for diagnosing OII [2]: detection of bacteria from peri-implant tissue and punction fluid (in joints), whereas swabs don’t play a role in diagnostics anymore [23]. False-negative results may occur in the case of prior antibiotic treatment [24, 25]. Three to five tissue samples should be taken from representative locations and incubated for up to ten days [5, 26, 27]. In the case of the presence of low-virulence pathogens, one single positive result may be interpreted as contamination [2, 5]. Multiplex-polymerase chain reaction (PCR) may offer new options but are not in extensive use until now [2, 28, 29]. Detection of the biofilm´s pathogens is promising and sonication of the explant is also used to detect not only planktonic bacteria as in tissue but metabolic inactive bacteria of matrix-secured biofilm but is expensive, not available in all hospitals and challenging in logistics for specimen transport [2, 30]. The best method for extraction and quantification of biofilm bacteria has not been found yet [31–34].

This study aimed to evaluate if pathogen-detection by culturing of explants with conventional microbiological methods and without sonication is superior to detection by culture of tissue and punction fluid. Finding a correlation between clinical infection and microbiological results using the methods described was not the aim of this study.

## Materials and methods

Implants were removed for different reasons such as infection, irritation, aseptic loosening. Removal of implant, harvesting of tissue from representative areas, and punction fluid in hip, knee, and shoulder joints was performed by the surgeon according to the study protocol. The hospital´s database was used for anonymized patient data, and comorbidities and other risk factors were evaluated.

**Fig. 1:**
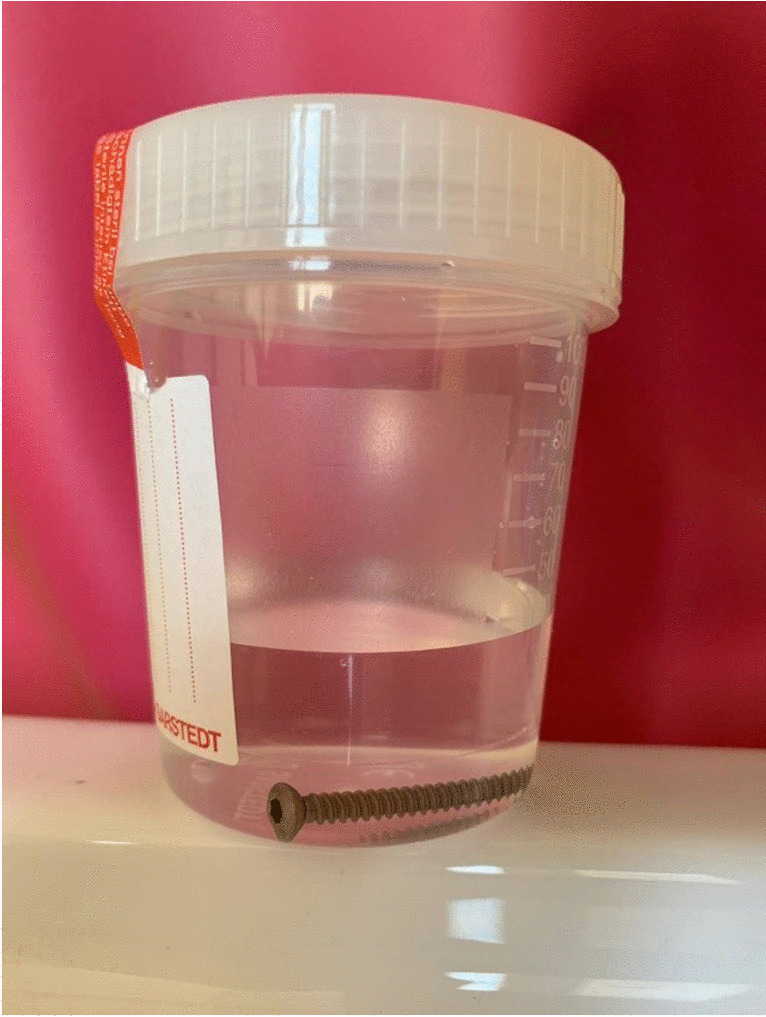
The sterile box for transport of explants (here: a screw)

The explant was put in a sterile tube (Falcon, Fisher Scientific, Fig. 1) and transferred to the microbiological institute within one hour. The sterile tube was filled with nutrient broth Schaedler Broth + Vit. K3 13 ml (bioMérieux SA) at the safety cabinet (HERASAFE2025, Thermo Scientific). The tube was transferred to the incubator HettCube 600 (Hettich) and incubated for ten days. The medium was checked daily and in case of haze seeded to an aerobe agar plate (BIO-RAD Chocolat agar PVS) and Schaedler-Agar (bioMérieux SA) for detection of anaerobe . If there was no haze after ten days, the fluid was seeded on the agars as described above and the aerobe agar was incubated for 48 hours in CO_2_-incubator HERAcell240i (Thermo Scientific), the anaerobe plate was placed in an Oxoid AnaeroGen 2.5 l bag (Thermo Scientific) with an Oxoid Resazurin-Anerobic-Indicator (Thermo scientific). In the case of bacterial growth, the bacterium was identified and its antibiotic sensitivity was determined using the automatic Vitek 2 (bioMérieux SA) system.

**Fig. 2:**
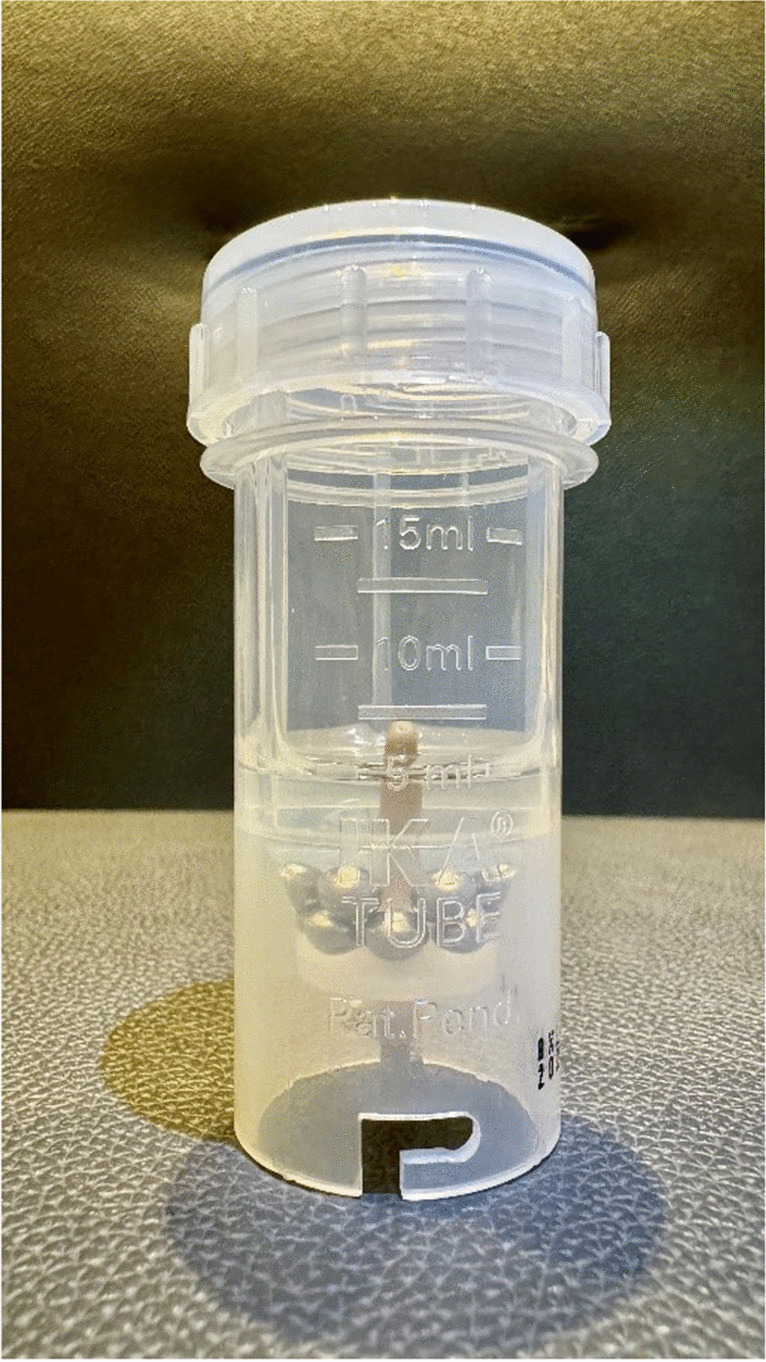
Box to grind and transport tissue samples

A tissue probe was placed in a sterile tube (ProbeAX, Axon Lab, Fig. 2) with NaCl and ten small steel balls to chop the tissue in the dispersant IKA ULTRA TURRAX (Tube Drive). The chopped tissue was struck on three aerobe agar plates (blood agar (Fisher Scientific), BIO-RAD Chocolat agar PVS, and Mac-Conkey-Agar (bioMérieux SA)) under a safety cabinet (HERASAFE2025). In addition, two anaerobe agar plates were used (Schaedler-Agar (bioMérieux SA) and Schaedler-Kanamycin-Vancomycin-Agar (bioMérieux SA)). The material was also placed in an anaerobe Schaedler Broth + vit. K3 nutrient broth (13 ml, bioMérieux SA), the rest was inoculated in an aerobe Brain Heart Infusion Broth (9 ml, bioMérieux SA). Blood agar and Mac-Conkey-Agar were incubated in a HettCube 600 (Hettich) for 24 hours. The chocolate agar was incubated in a CO_2_-incubator HERAcell240i (Thermo Scientific). After 24 hours, aerobe agar plate growth was evaluated. In the case of growth, the bacterium was identified and its antibiotic sensitivity determined. Mac-Conkey-agar was discarded if there was no growth after 24 hours. Chocolate agar was incubated for 10 days in the CO_2_-incubator HERAcell240i (Thermo Scientific) and the case of no growth discarded. Identification of bacteria and antibiotic sensitivity testing was done with Vitek 2 (bioMérieux). Anaerobe agar plates were put in 2.5-liter boxes filled with Oxoid AnaeroGen and Oxoid Resazurin-Anaerobe-Indicator (Thermo Scientific) and checked after 48 hours, four, seven and ten days. Aerobe plates were checked after 24 hours, 48 hours, four, seven and ten days. In case of no bacterial growth after ten days, a final seed in a Schaedler- and chocolate agar was done for 48 hours.

The punction of fluid was transported in a Falcon tube (Fisher Scientific) and centrifugated using Heraeus Megafuge 16 (Thermo Scientific). With the sediment, a gram stain was performed using Previ Color Gram (bioMérieux). Microscopic observation of the Gram preparations was carried out using a Nikon Eclipse Ci microscope. Parallel to the preparation of each Gram preparation, parts of the punctate were inoculated onto three aerobic plates under the safety cabinet (HERASAFE2025, Thermo Scientific): blood agar (Fisher Scientific), chocolate agar (BIO-RAD Chocolat agar PVS) and Mac-Conkey agar (bioMérieux SA). Two more anaerobic agar plates were added: Schädler agar (bioMérieux SA) and Schaedler kanamycin-vancomycin agar (bioMérieux SA). Another part of the punctate was added to an anaerobic Schaedler Broth + vit. K3 nutrient broth (13 ml, bioMérieux SA) and another part was transferred to an aerobic liquid medium: Brain Heart Infusion Broth (9 ml, bioMérieux SA). The blood agar and Mac-Conkey agar were placed in the HettCube 600 incubator (Hettich) for 24 hours. The chocolate agar was placed in the HERAcell240i CO_2_ incubator (Thermo Scientific) from the beginning. All aerobic agar plates were read after 24 hours. If aerobic germs had already grown after this period, bacteria were identified and their antibiotic sensitivity determined. The Mac-Conkey agar plate was discarded if no growth was observed after 24 hours. If no growth was seen on the blood agar plate, this plate was also placed in the HERAcell240i CO_2_ incubator. The blood agar and the cooked blood agar were then incubated in the CO_2_ incubator for up to ten days. If germs had grown during this period, this was also followed by identification and resistance testing. The anaerobic agar plates were incubated as described above. The aerobic plates of the analyzed punction fluids were also checked regularly: After 24 hours, 48 hours, four days, seven days, and ten days respectively. The anaerobic plates were examined for the first time after 48 hours, then also after four days, seven days, and ten days - in the same way as the biopsy specimens. If microbial growth was detected at any of these points in time, bacteria were identified and their antibiotic sensitivity determined as described above.

To rule out contamination of the sample containers, 19 empty sample containers were explored for bacterial growth. No bacterial growth was detected in any of these sample containers. This ensured that bacteria could only be detected by the respective sample material, i.e. from the metal explant, the tissue sample, or the punctate.

We obtained a positive vote from the local ethical committee (2018 618N MA).

Statistical analysis was performed with the help of the Institute of Medical Statistics/Heinrich-Lanz-Center of Medical Faculty Mannheim with the use of Excel® 2019 (Microsoft) and SAS (Version 9.4 (SAS Institute, Inc., Cary, North Carolina). Shapiro-Wilk-test was used for age distribution, comparison of two different groups was performed with the Mann-Whitney-U-test. More groups were assessed using the Kruskal-Wallis test. The chi^2^-test was used for qualitative testing, exact Fisher-test for calculation of contingency tables and significance. α = 0.05 was determined as significant.

## Results

**Fig. 3:**
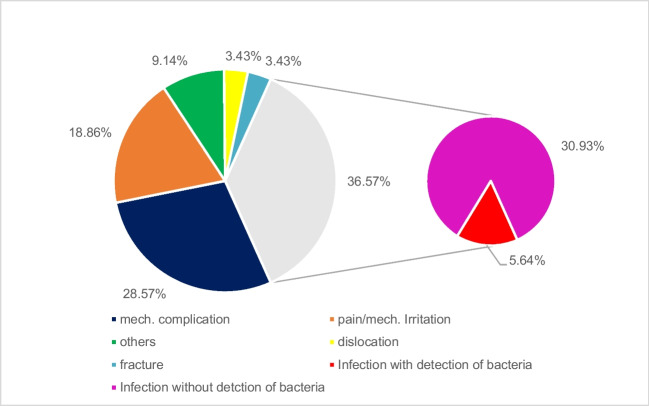
Indication for implant removal

Between February 2018 and April 2019, 175 consecutive cases (163 patients, 89 females, 86 males, mean age 62.1 years, range 16 to 89) were included in this prospective study with the removal of orthopaedic implants in a single university trauma centre in Germany. Indication for implant removal was an infection in 64 cases (36.6%), loose implant in 50 cases (28.6%), irritation and painful implant in 33 cases (18.9%), and patient´s wish in 16 cases (9.1%) (Fig. 3). 52 patients (29.7%) had been treated with implant-related infection before removal, and another 29 patients (16.6%) needed inpatient treatment for infection. Patients showed several comorbidities: 97 patients (55.4%) had hypertonia, 35 patients (20%) had coronary disease, and 23 patients (13.1%) had diabetes as the main comorbidities. Tumour disease (13.1%), kidney failure (10.9%), obesity (6.9%), osteoporosis (6.3%), and rheumatoid disease (4.6%) were other comorbidities identified. Most patients had more than one comorbidity. 113 patients (64.6%) had no signs of infection in blood cell count (CRP <5mg/l and white blood cells <10000/µl) at the time of surgery. 89 implants (51.4%) have been removed from the femoral region, 42 (24.3%) from the lower leg region, and 18 (10%) in the spine region. ten implants (5.8%) were removed from the humerus, eight (4.6%) from the forearm and six (3.5%) from the pelvis. In two cases, the exact region was unclear due to unclear documentation of removal between the two regions. The authors decided to define the region according to the surgical report. In 148 cases (84.6%), no bacteria could be detected before implant removal, in 61 cases (34.9%), antibiotic treatment was administered before surgery (Table 1). 25 patients (14.3%) had one revision surgery before implant removal, and another 32 patients (18.3%) had two or more surgeries. 30 (17.1%) of 175 specimens could not be fully evaluated according to the study protocol (155 cases with tissue/punction fluid, 165 cases with explants): in ten cases, the transport box of the explant was damaged and in 20 cases, punction fluid was not enough for evaluation or not taken so the comparison was impossible. Overall, 145 cases were included in the study with explant, tissue, and punction fluid.

**Table 1:** Shows better diagnostic performance of explant´s surface in the case of antibiotic therapy prior to surgery

	antibiotic therapy prior implant removal	no antibiotic therapy	total	p-value
biopsy/punction fluid detected bacteria	41 (26.45%)	23 (14.84%)	64 (41.29%)	<0.0001
biopsy/punction fluid no detected bacteria	18 (11.61%)	73 (47.10%)	91 (58.71%)	
Explant’s surface detected bacteria	38 (23.03%)	58 (35.15%)	96 (58.18%)	0.0709
Explant’s surface no detected bacteria	18 (10.91%)	51 (30.91%)	69 (41.82%)	

**Fig. 4:**
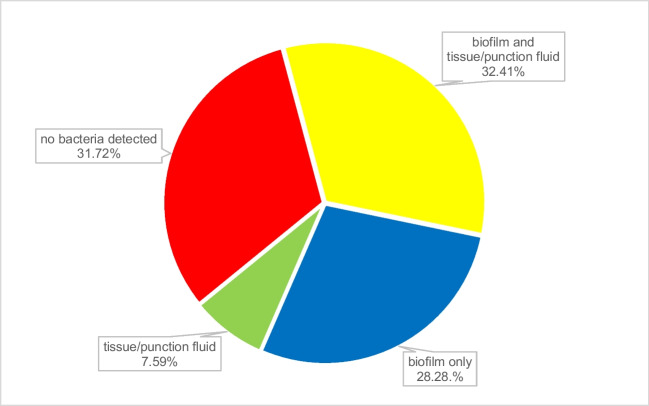
Findings related to used method

In 46 cases (31.7%), no bacteria could be detected. 71 of 145 cases (49.0%) showed the same bacteria in both tissue and explant´s surface. With tissue and punction fluid alone, bacteria could be detected in 91 cases (62.8%), on explant´s surface alone, bacteria could be detected in 96 cases (66.2%, p<0.001, tissue/punction fluid compared to explant´s surface). In 41 cases (28.3%), bacteria could only be detected from the explant´s surface whereas this was only the case in 11 cases (7.6%) of tissue/punction fluid (Fig. 4, p<0.001, explant´s surface compared to tissue/punction fluid). In another 22 cases, additional bacteria could be detected from explant´s surface or tissue/punction fluid (11 and 11, respectively). 25 cases (36.5%) showed same bacteria throughout all examination methods: in 123 cases (71+41+11 = 84.83%), detection of pathogens from explant´s surface was equal or superior compared to tissue and punction fluid (93 cases, 71+11+11 = 64.14%, p<0.0001, explant´s surface compared to tissue/punction fluid).

**Fig. 5:**
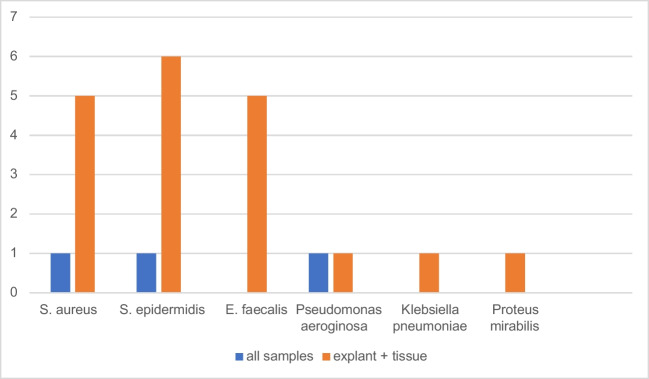
Most common bacteria detected by all methods and explant´s surface/tissue

**Fig. 6:**
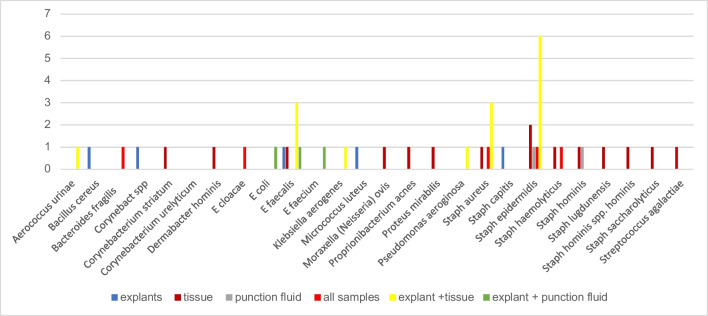
Shows only the additional detected organisms unraveled by the method

**Fig. 7:**
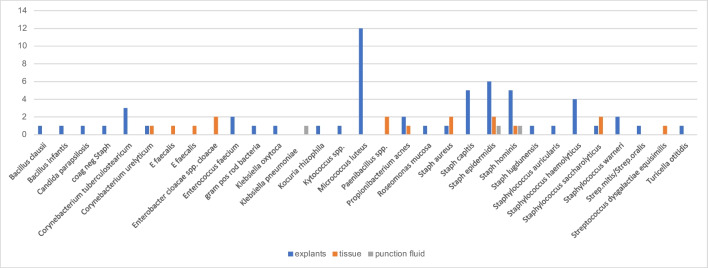
Shows the single detected organisms particular by each method

The spectrum of organisms detected is provided in Table 2. *Staphylococcus epidermidis, S. aureus and E. faecalis* were most commonly detected by all diagnostic methods used (Fig. 5). Figure 6 shows only the additional detected organisms unraveled by the method used in patients with multiple bacteria detected, Fig. 7 shows the single detected organisms particular by each method. Age, sex, reason for surgery, type of implant or hypertonia, and original diagnosis for implantation or other comorbidities did not show statistically significant influences on results (p>0.05, respectively). In cases with or without antibiotic treatment before surgery, bacteria could be detected in tissue/Punction fluid in 11.6% and 14.8% in cases without antibiotic treatment (Table 1, p=0.0001). In cases with or without antibiotic treatment before surgery, bacteria could be detected in explant´s surface in 23.0% and 35.2% (p=0.071). In this collective, antibiotic treatment before surgery had a statistically significant influence of detection of bacteria of tissue or punction fluid but not of explant´s surface. The differences in detection between tissue/punction fluid and explants´s surfacewere statistically significant (p=0.0001).

**Table 2:** Shows the spectrum of all organisms detected

High-Virulence organisms	Multidrug-resistant organisms	Low-Virulence organisms
Enterobacter cloacae	Methicillin-resistant Staphylococcus aureus	Bacillus cereus
Enterococcus faecalis		Bacillus clausii
Enterococcus faecium		Candida parapsilosis
Escherichia coli		Corynebacterium striatum
Klebsiella aerogenes		Corynebacterium urelyticum
Klebsiella pneumoniae		Corynebacterium spp.
Proteus mirabilis		Dermabacter hominis
Pseudomonas aeruginosa		Micrococcus luteus
Staphylococcus aureus		Paenibacillus spp.
Streptococcus agalactiae		Propionibacterium acnes
Streptococcus dysgalacticae equisimilis		Staphylococcus capitis
		Staphylococcus epidermidis
		Staphylococcus haemolyticus
		Staphylococcus hominis spp. hominis
		Staphylococcus saccharolyticus
		Staphylococcus warneri
		Stenotrophomonas maltophilia
		Turicella otitides

## Discussion

In this study, the detection of bacteria of orthopaedic explant´s surface showed significantly higher sensitivity (84.8%) compared to tissues or punction fluid (64.1%). Treatment of orthopaedic implant-related infections is challenging for both patient and surgeon as well as expensive [3]. Fast and correct detection of bacteria causing the infection is crucial for correct treatment, independent of age or comorbidities [9, 12, 18, 35].

 Reasons for the implantation and explantation of orthopaedic implants were multifaceted, similar to other studies [5]. No differences in the detection of bacteria were seen regarding comorbidities [12–18, 36–43]. A broad range of pathogens could be identified, the spectrum was similar to other studies: In a study of Corvec et al., coagulase-negative Staphylococci were identified as the most common pathogens in OII [5, 44]. Multiple bacteria are present from 10 to 30%, revision surgery and longer operation times are suspected main reasons [6, 19]. In our study, *Corynebacterium* was identified as a rare pathogen in OII, similar to Holinka et al. [45]. *Micrococcus luteus* and *Stenotrophomonas maltophilia* were detected quite often in the explant´s biofilm. The presence of these was described before in patients with rheumatoid disease and immune-suppressive therapy [46]. *Micrococcus luteus* was described as a non-pathogenic bacteria but not previously discussed as a pathogen in OII. It remains uncertain if the presence of *Micrococcus luteus* on the implant´s surface has any clinical impact. No pathogens could be detected in many patients in our collective because these patients received orthopedic implant removal for other reasons than infection. Linking clinical findings and the presence of infection to microbiological diagnostic methods was not the aim of this study. Font-Vizcarra et al. showed that swabs are not suitable for OII diagnostics, their only advantage is the low cost [23, 28, 47]. Trampuz et al. compared the diagnostics of sonication fluid with tissue and showed higher sensitivity compared to tissue (79%, and 61%, respectively) [28, 30]. Because of costs and the complexity of transport and procedure, sonication is not available in all hospitals [29, 47]. Portillo et al. showed higher sensitivity of multiplex-PCR compared to sonication but this procedure is also expensive and did not become standard procedure so far as there is a high risk of contamination and false positive results [48]. In a prior study, the author of this study showed a high negative but low positive predictive value in OII – diagnostics using multiplex-PCR [49]. Today, the gold standard for OII diagnostics remains three to five tissue samples and conventional microbiological methods [2]. More samples could lead to more false-positive results because of contamination [50]. Reasons for negative results may be prolonged transport to the microbiological lab, too short incubation time, prior antibiotic therapy, or low-virulent pathogens [7]. In our study, all samples were transported immediately within hours to the lab and all samples were incubated for ten days. Low-virulent and slow-growing pathogens can be detected safely using this technique [6]. Schäfer et al. described only 74% of bacteria detection in the first seven days, and other bacteria up to ten days, so we decided on ten days in our study [25]. We could see a statistical significance of prior antibiotic therapy and diagnostic results of tissue and punction fluid in our collective, similar to Flesch et al. [7]. Our results support the theory that biofilm diagnostic is superior to tissue. In 35.9%, we detected more pathogens in biofilm compared to tissue and punction fluid, which was seen before [2]. One reason may be the biofilm protection for antibiotic therapy which is not present in tissue samples [2, 21, 22]. Antibiotic treatment should be initiated immediately in case of acute infection and not delayed until samples can be taken which is not recommended in chronic infection [19, 25, 51]. In 15.2% (22 cases), pathogens could only be shown in tissue or punction fluid. Contamination with skin or absence of biofilm may be the main reason for this as published before [30]. In 84.8%, the explant´s surface showed pathogens – in 64.1%, tissue and punction fluid showed pathogens - in 49.0%, both explant´s surface and tissue showed pathogens (p<0.001, respectively) which supports our hypothesis that diagnostic of explant´s surface fluid is superior to tissue and/or punction fluid. Instead of up to five samples, one sample of explant´s surface may be enough for safe and correct diagnostics which reduces costs and spares resources [4].

This study has several limitations: First, this study was performed in a single centre in Germany. An international multiple-centre study could lead to the detection of different pathogens and different results. Second, the study sample was relatively small, a larger sample size could also lead to different results. Third, co-morbidities were evaluated according to electronic patient data: missing or wrong information may have influenced our results with no statistically significant influence of any co-morbidity. Fourth, no comparison to other methods such as sonication or PCR and no correlation to clinical infection was performed: this could be the topic of the next study in a multi-centric setting. Fifth, employees of the microbiological institute knew about this study – this may have biased the results or the methods of their work but this was mandatory due to the ethical committee vote. An advantage was the diagnostics at one single center with the same procedures and machines. Sixth, the pooling of five tissue samples may also reduce costs but would not identify as many pathogens as explant´s surface. Lastly, only English and German publications were included in the literature research.

In summary, our study supports the hypothesis that pathogen diagnosis of orthopaedic explant’s surface using incubation of the transport fluid is superior compared to tissue samples. Now that this method is proven to be functional, cheap, and safe, future multiple-centre studies considering the clinical status as well may support the findings of our present study.

## Data Availability

No datasets were generated or analysed during the current study.
